# The Impact of Control Interventions on Malaria Burden in Young Children in a Historically High-Transmission District of Uganda: A Pooled Analysis of Cohort Studies from 2007 to 2018

**DOI:** 10.4269/ajtmh.20-0100

**Published:** 2020-05-18

**Authors:** Moses R. Kamya, Abel Kakuru, Mary Muhindo, Emmanuel Arinaitwe, Joaniter I. Nankabirwa, John Rek, Victor Bigira, James Kapisi, Humphrey Wanzira, Jane Achan, Paul Natureeba, Anne Gasasira, Diane Havlir, Prasanna Jagannathan, Philip J. Rosenthal, Isabel Rodriguez-Barraquer, Grant Dorsey

**Affiliations:** 1 Department of Medicine, Makerere University, Kampala, Uganda;; 2 Infectious Diseases Research Collaboration, Kampala, Uganda;; 3 Uganda National Health Laboratory Services, Kampala, Uganda;; 4 Pilgrim Africa, Kampala, Uganda;; 5 Medical Research Council Unit, Banjul, The Gambia;; 6 Makerere University-John Hopkins University Collaboration, Kampala, Uganda;; 7 Department of Medicine, University of California San Francisco, San Francisco, California;; 8 Department of Medicine, Stanford University, Stanford, California

## Abstract

There is limited evidence on whether malaria elimination is feasible in high-transmission areas of Africa. Between 2007 and 2018, we measured the impact of malaria control interventions in young children enrolled in three clinical trials and two observational studies in Tororo, Uganda, a historically high-transmission area. Data were pooled from children aged 0.5–2 years. Interventions included individually assigned chemoprevention and repeated rounds of indoor residual spraying (IRS) of insecticide. All children received long-lasting insecticidal nets (LLINs) and treatment for symptomatic malaria with artemisinin-based combination therapy. Malaria incidence was measured using passive surveillance and parasite prevalence by microscopy and molecular methods at regular intervals. Poisson’s generalized linear mixed-effects models were used to estimate the impact of various control interventions. In total, 939 children were followed over 1,221.7 person years. In the absence of chemoprevention and IRS (reference group), malaria incidence was 4.94 episodes per person year and parasite prevalence 47.3%. Compared with the reference group, implementation of IRS was associated with a 97.6% decrease (95% CI: 93.3–99.1%, *P* = 0.001) in the incidence of malaria and a 96.0% decrease (95% CI: 91.3–98.2%, *P* < 0.001) in parasite prevalence (both measured after the fifth and sixth rounds of IRS). The addition of chemoprevention with monthly dihydroartemisinin–piperaquine to IRS was associated with a 99.5% decrease (95% CI: 98.6–99.9%, *P* < 0.001) in the incidence of malaria. In a historically high–malaria burden area of Uganda, a combination of LLINs, effective case management, IRS, and chemoprevention was associated with almost complete elimination of malaria in young children.

## INTRODUCTION

Significant progress in malaria control has been realized over the last 15 years, with declines in malaria burden such that many countries are now targeting elimination.^1–4^ However, global progress in reducing the incidence of malaria has flattened in recent years, and malaria is on the rise across many high-burden African countries.^4^ In sub-Saharan Africa, progress toward pre-elimination or elimination has primarily been limited to relatively low-transmission settings.^5–7^ Whether malaria elimination will be feasible in the coming decades in historically high-transmission areas of sub-Saharan Africa given available interventions remains unclear.^8,9^ Indeed, elimination in high-transmission areas presents particular challenges given the very large number of infectious vectors and the large number of infected people, a majority of whom are asymptomatic.^8,10^

Uganda is one of the highest malaria burden countries in the world. Control interventions in Uganda have included treatment of uncomplicated malaria with artemether–lumefantrine (AL) since 2005, universal distribution of long-lasting insecticidal nets (LLINs) in 2013–2014 and 2017–2018, and indoor residual spraying (IRS) of highly effective insecticides in selected districts since 2006. With the scale-up of these control interventions, there has been evidence of a significant decrease in the burden of malaria over the last decade.^11–13^ However, these gains have been fragile, with resurgence observed following the cessation of key interventions.^14^

Beginning in 2007, we have measured the impact of various malaria control interventions in Tororo, Uganda, a historically high-transmission area,^15^ using cohorts of children enrolled in three clinical trials^16–19^ and two observational studies.^12,15^ In these studies, interventions have included various individually assigned chemoprevention regimens and repeated rounds of IRS of insecticide starting in December 2014. Here, we use pooled analysis to estimate the impact of this combination of interventions on the malaria burden in Tororo over time.

## MATERIALS AND METHODS

### Study setting.

All studies were conducted between August 2007 and December 2018 in Tororo district, Uganda. Malaria transmission intensity has been historically high in Tororo, with an entomological inoculation rate estimated at 310 infective bites per person per year in 2011–2012.^15^ Before the implementation of IRS in 2014 onward, malaria transmission in Tororo was perennial, with minor peaks after the two rainy seasons in April–June and September–December. After implementation of IRS, transmission declined markedly, with peaks generally occurring just before each round of IRS. Before implementation of IRS, *Anopheles gambiae* sensu stricto was the major vector, but following IRS implementation, *Anopheles arabiensis* became the dominant species.^20^ Pyrethroid resistance in the area is common, with resistance of *A. gambiae* to deltamethrin and permethrin reported to be 82% and 67%, respectively; resistance to bendiocarb was not observed.^21^ Before November 2013, malaria control in Tororo was limited to the distribution of LLINs through antenatal care services, promotion of intermittent preventive treatment with SP during pregnancy, and treatment of uncomplicated malaria with AL.

### Study design, participants, and individual-level chemoprevention.

We conducted a secondary analysis of pooled data from three clinical trials, the Tororo Child Cohort (TCC) study,^16,19^ the Prevention of Malaria and HIV Disease in Tororo (PROMOTE) Project 3,^17^ and Birth Cohort 1^18^ studies, and two observational studies from the Program for Resistance, Immunology, Surveillance & Modeling of Malaria in Uganda (PRISM), PRISM 1^12,15^ and PRISM 2 (Table 1). Details of each study have been described previously. In brief, the TCC study used convenience sampling to enroll 100 children aged between 6 weeks and 12 months born to HIV-uninfected mothers. Children were randomized to treatment with AL or dihydroartemisinin–piperaquine (DP) for all episodes of uncomplicated malaria and followed up to 5 years. The PROMOTE Project 3 study used convenience sampling to enroll 400 children aged between 4 and 6 months born to HIV-uninfected mothers. Children were randomized to no chemoprevention, daily trimethoprim–sulfamethoxazole (TS), monthly sulfadoxine-pyrimethamine (SP) (single dose), or monthly DP (once a day for 3 days) given unsupervised at home from 6 months to 2 years of age, and then followed up for one additional year after chemoprevention was stopped. The PROMOTE Birth Cohort 1 study included a birth cohort of 291 children born to HIV-uninfected mothers. Children were randomized to receive DP (only the first day of each 3-day treatment was directly observed) every 12 weeks or every 4 weeks from 6 months to 2 years of age and then followed up for one additional year after chemoprevention was stopped. The PRISM 1 study enrolled 364 children aged between 6 months and 10 years from 107 households randomly selected from within Nagongera subcounty of Tororo district. Children were not randomized to any interventions and followed up to 6 years. The PRISM 2 study enrolled 497 participants of all ages from 80 households randomly selected from within Nagongera subcounty. Participants were not randomized to any interventions and followed up for up to 14 months. Children were not enrolled in more than one study.

**Table 1:** Characteristics of cohort studies and participants stratified by intervention groups

Study	Dates of observation	Intervention group		Number of children	Mean age, years (SD)	Female (%)	Person years of observation	Number of routine visits
		Chemoprevention	IRS					
Tororo Child Cohort	August 7–September 9	None	None	98	1.3 (0.4)	40 (40.8%)	135.0	1,405
PROMOTE Project 3	July 10–September 12	None	None	98	1.2 (0.4)	48 (49.0%)	138.0	1,418
		Daily TS unobserved		99	1.2 (0.4)	48 (48.5%)	140.0	1,431
		Monthly SP unobserved		98	1.2 (0.4)	44 (44.9%)	135.5	1,346
		Monthly DP unobserved		98	1.2 (0.4)	52 (53.1%)	135.0	1,284
PRISM 1	August 11–January 15	None	None	92*	1.4 (0.4)	45 (48.9%)	83.0	432
	February 15–September 17	None	Rounds 1–4	44*	1.6 (0.3)	24 (54.6%)	25.6	234
PROMOTE Birth Cohort 1	April 15–May 17	DP observed every 12 weeks	Rounds 1–3	183	1.2 (0.4)	92 (50.3%)	262.5	3,486
		DP observed every 4 weeks		93	1.2 (0.4)	44 (47.3%)	135.6	1,809
PRISM 2	October 17–December 18	None	Rounds 5–6	51	1.4 (0.4)	24 (47.1%)	31.5	424

DP = dihydroartemisinin–piperaquine; IRS = indoor residual spraying; PROMOTE = Prevention of Malaria and HIV Disease in Tororo.

* One hundred twenty-one unique children included from the PRISM 1 study: 63 only observed before IRS implemented, 29 observed before and after IRS implemented, 15 observed only after IRS implemented.

### Study procedures and follow-up of study participants.

All study participants received an LLIN at enrolment and followed up in dedicated study clinics for all their medical care. Routine visits were conducted once a month, except for the PRISM 1 study, in which routine visits were conducted every 1–3 months. Routine visits included collection of blood for assessment of parasitemia by microscopy in all studies using a rigorous quality control system. In the PRISM 1 and PROMOTE Birth Cohort 1 studies, dried blood spots were collected at the time of routine visits for loop-mediated isothermal amplification (LAMP) to detect *Plasmodium falciparum* as previously described.^22^ In the PRISM 2 study, whole blood was collected at the time of routine visits for an ultrasensitive quantitative PCR (qPCR) assay to detect *P. falciparum* with a lower limit of detection of 0.06–0.15 parasites per microliter as previously described.^23^ In all studies, participants with asymptomatic parasitemia were not provided antimalarial treatment, in accordance with local guidelines. Parents/guardians were encouraged to bring their children to the study clinics any time they were ill and reimbursed for their transport costs. Children who presented with a recent history of fever or a tympanic temperature of ≥ 38.0°C had a thick blood smear performed and those positive by microscopy diagnosed with malaria. Uncomplicated malaria was treated with AL and complicated malaria with quinine or IV artesunate in all studies except for the TCC study, in which uncomplicated malaria was treated with AL or DP, depending on the assigned regimen for each study subject.

### Population-level vector control interventions.

In addition to all study participants receiving an LLIN at enrollment, universal LLIN distribution campaigns targeting one LLIN per every two persons were conducted in Tororo district in November 2013 and May 2017. Indoor residual spraying using carbamate bendiocarb was carried out in Tororo district in December 2014–February 2015, June–July 2015, and November–December 2015. Indoor residual spraying with pirimiphos-methyl (Actellic), a long-lasting organophosphate, was carried out in July 2016, June–July 2017, and June–July 2018. Overall IRS coverage in Tororo district was 85% for the first round and over 90% for subsequent rounds. For households participating in the PRISM cohort studies, IRS coverage ranged from 93 to 100%.

### Statistical methods.

Individual-level data from all studies were pooled and analyzed using Stata version 14.2 Stata Corporation, College Station, TX) and R version 3.3.2 (R Core Team, 2016, Vienna, Austria). Analyses included only observation time for children aged between 0.5 and 2 years, as this age range was represented in all studies. The intervention groups assessed included no chemoprevention and no IRS (reference group), daily TS unobserved with no IRS, monthly SP unobserved with no IRS, monthly DP unobserved with no IRS, no chemoprevention with rounds 1–4 of IRS, DP observed every 12 weeks with rounds 1–3 of IRS, DP observed every 4 weeks with rounds 1–3 of IRS, and no chemoprevention with rounds 5–6 of IRS (Table 1). Three of the studies (TCC, PROMOTE Project 3, and PRISM 1) included children who did not receive chemoprevention before IRS was implemented, one study (PROMOTE Project 3) included children who received chemoprevention before IRS was implemented, one study (PROMOTE Birth Cohort 1) included children who received chemoprevention after IRS was implemented, and two studies (PRISM 1 and 2) included children who did not receive chemoprevention after IRS was implemented (Table 1). Outcomes evaluated included the incidence of symptomatic malaria and prevalence of microscopic parasitemia. In addition, the prevalence of microscopic or submicroscopic parasitemia (as assessed by LAMP or qPCR) was evaluated in three of the studies. Associations between intervention groups and outcomes were estimated using Poisson’s generalized linear mixed-effects models including random effects to account for clustering within individuals and adjusted for study and age. Associations between intervention groups and the incidence of symptomatic malaria were expressed as the protective efficacy defined as 1—the incidence rate ratio (incidence in the intervention group/incidence in the reference group). Associations between intervention groups and measures of parasite prevalence were expressed as the relative reduction defined as 1—prevalence ratio (prevalence in the intervention group/prevalence in the reference group).

### Ethical approval.

All studies were approved by the ethics committees of Makerere University, the Uganda National Council for Science and Technology, and the University of California, San Francisco. Written informed consent was provided by parents/guardians for all study participants.

### Role of the funding source.

The funders played no role in the design of the study; in the collection, analyses, and interpretation of data; in the writing of the manuscript; or in the decision to submit the manuscript for publication.

## RESULTS

### Characteristics of the study participants.

**Figure 1.**
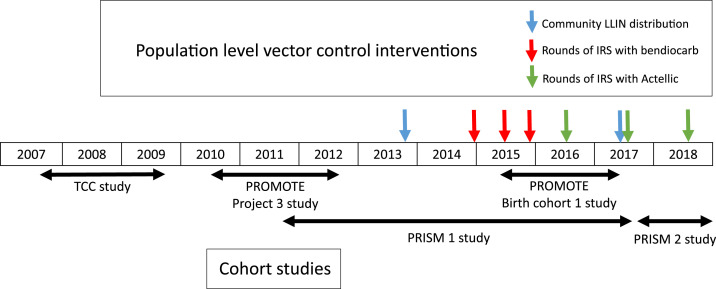
Timeline of when the various cohort studies were conducted and when the population-level vector control interventions were implemented. This figure appears in color at www.ajtmh.org.

Study participants resided in houses located throughout the central and eastern portion of Tororo district, with the exception of PRISM 1 and 2 study participants, who resided in Nagongera subcounty in central Tororo. A few participants, primarily from the TCC and PROMOTE Project 3 studies, lived outside the borders of Tororo district. Figure 1 shows the timeline of when the various cohort studies were conducted and when the population-level vector control interventions were implemented. Observations contributing to the analyses extended over 11 years, from August 2007 to December 2018, with continuous overlapping observation time, with the exception of a gap between October 2009 and June 2010 (Table 1, Figure 1). A total of 939 children were observed between 6 months and 2 years of age, resulting in 1,221.7 person years of follow-up and 13,269 routine visits in which parasitemia was assessed. The mean age of study participants during the follow-up was 1.3 years, and 48.2% were female. Only two children died (both due to pneumonia), one from the PROMOTE Project 3 study who was not receiving chemoprevention before IRS was implemented and one from the PROMOTE Birth Cohort 1 study who was receiving DP every 12 weeks after IRS was implemented.

### Association between chemoprevention, IRS, and the incidence of symptomatic malaria.

**Figure 2.**
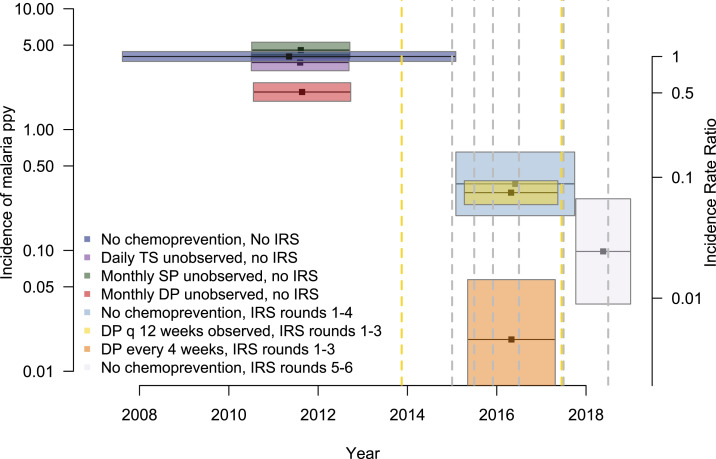
Adjusted incidence of malaria among participants from each intervention group. Each intervention is represented by a distinct colored box. The length of the boxes indicates the duration of the studies, and width indicates the 95% CI of the estimate. Incidence rate ratios, relative to the reference group (no chemoprevention, no indoor residual spraying [IRS]), are shown as a secondary *y*-axis. Vertical dashed lines indicate the timing of population-level interventions: long-lasting insecticidal nets distribution (yellow) and rounds of IRS (gray). This figure appears in color at www.ajtmh.org.

Among children who did not receive chemoprevention before the implementation of IRS (reference group), the crude and age-adjusted incidence of symptomatic malaria were 4.94 and 4.03 episodes per person year, respectively (Table 2). Chemoprevention with daily TS or monthly SP given unobserved at home was not associated with any significant protection against malaria. Chemoprevention with monthly DP given unobserved at home was associated with a protective efficacy of 49.2% (95% CI: 38.2–58.2%, *P* < 0.001). In children who were not receiving chemoprevention, the first four rounds of IRS were associated with a protective efficacy of 91.2% (95% CI: 83.7–95.2%, *P* < 0.001), while the fifth and sixth rounds of IRS were associated with a protective efficacy of 97.6% (95% CI: 93.3–99.1%, *P* = 0.001). During the first three rounds of IRS, chemoprevention with DP given every 12 weeks, with the first daily dose observed in the clinic, was not associated with additional protective efficacy as compared with IRS alone (protective efficacy of 92.5%, 95% CI: 90.5–94.1%, *P* < 0.001); by contrast, DP given every 4 weeks was associated with almost complete protection against malaria (protective efficacy 99.5%, 95% CI: 98.6–99.9%, *P* < 0.001) (Table 2, Figure 2). Among 3,580 total episodes of symptomatic malaria, only 13 (0.4%) met the criteria for severe malaria (repeated convulsions = 8, severe anemia = 4, and cerebral malaria = 1), and there were no deaths due to malaria.

**Table 2:** Associations between intervention groups and the incidence of symptomatic malaria

Intervention group		Episodes of malaria	Person years of follow-up	Crude incidence*	Adjusted incidence†	Protective efficacy‡ (95% CI)	*P*-value
Chemoprevention	Indoor residual spraying						
None	None	1,757	356.0	4.94	4.03	reference group	
Daily trimethoprim–sulfamethoxazole unobserved		614	140.0	4.39	3.59	10.9% (−6.5–25.5%)	0.20
Monthly SP unobserved		730	135.5	5.39	4.55	−12.9% (−34.6–5.3%)	0.18
Monthly DP unobserved		361	135.0	2.67	2.05	49.2% (38.2–58.2%)	< 0.001
None	Rounds 1–4	12	25.6	0.47	0.36	91.2% (83.7–95.2%)	< 0.001
DP observed every 12 weeks	Rounds 1–3	99	262.5	0.38	0.30	92.5% (90.5–94.1%)	< 0.001
DP observed every 4 weeks		3	135.6	0.02	0.02	99.5% (98.6–99.9%)	< 0.001
None	Rounds 5–6	4	31.5	0.13	0.10	97.6% (93.3–99.1%)	0.001

DP = dihydroartemisinin–piperaquine.

* Per person year.

† Adjusted incidence per person year for a child of average age (1.25 years).

‡ Including random effects to account for clustering within individuals and adjusted for study and age.

### Association between chemoprevention, IRS, and the prevalence of microscopic parasitemia.

**Figure 3.**
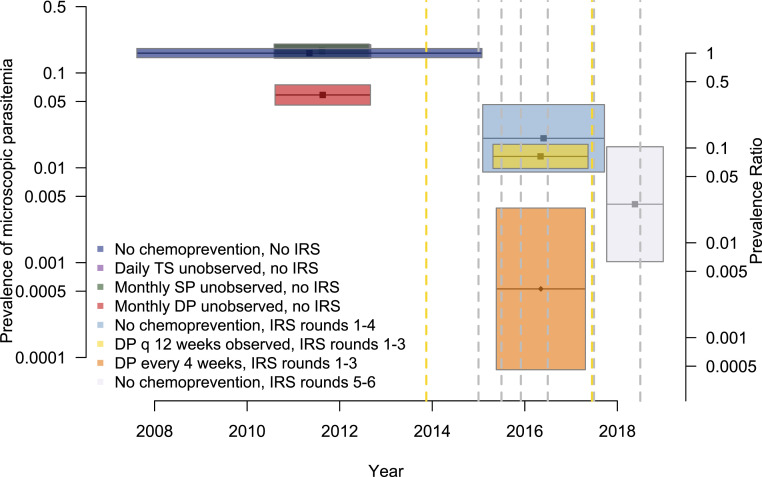
Adjusted prevalence of microscopic parasitemia among participants from each intervention group. Each intervention is represented by a distinct colored box. The length of the boxes indicates the duration of the studies, and width indicates the 95% CI of the estimate. Prevalence ratios, relative to the reference group (no chemoprevention, no indoor residual spraying [IRS]), are shown as a secondary *y*-axis. Vertical dashed lines indicate the timing of population-level interventions: long-lasting insecticidal nets distribution (yellow) and rounds of IRS (gray). This figure appears in color at www.ajtmh.org.

Among children who did not receive chemoprevention before the implementation of IRS (reference group), the crude and age-adjusted prevalence of microscopic parasitemia at the time of routine visits were 17.6% and 16.2%, respectively (Table 3). The effects of interventions on the prevalence of microscopic parasitemia were broadly consistent with the effects on incidence. There were no reductions in prevalence among children receiving unsupervised chemoprevention with daily TS or monthly SP, whereas monthly DP was associated a relative reduction of 63.8% (95% CI: 52.9–72.2%, *P* < 0.001). The first four rounds of IRS were associated with a relative reduction of 87.4% (95% CI: 71.2–94.5%, *P* < 0.001) in children not receiving chemoprevention, and this effect increased to 97.4% (95% CI: 89.7–99.4%, *P* < 0.001) during the fifth and sixth rounds of IRS. Similar to the incidence results, adding chemoprevention with DP given every 12 weeks did not provide additional benefits during the first three rounds of IRS (relative reduction of 91.9%, 95% CI: 88.9–94.0%, *P* < 0.001), although DP given every 4 week was associated with almost complete protection against microscopic parasitemia (protective efficacy 99.7%, 95% CI: 97.7–100%, *P* < 0.001) (Table 3, Figure 3).

**Table 3:** Associations between intervention groups and the prevalence of microscopic parasitemia

Intervention group		Crude prevalence	Adjusted prevalence*	Relative reduction† (95% CI)	*P*-value
Chemoprevention	Indoor residual spraying				
None	None	573/3,255 (17.6%)	16.2%	reference group	
Daily trimethoprim–sulfamethoxazole unobserved		265/1,431 (18.5%)	16.8%	−4.1% (−25.8–13.9%)	0.68
Monthly SP unobserved		253/1,346 (18.8%)	17.1%	−5.5% (−27.8–12.9%)	0.58
Monthly DP unobserved		82/1,284 (6.4%)	5.9%	63.8% (52.9–72.2%)	< 0.001
None	Rounds 1–4	6/234 (2.6%)	2.0%	87.4% (71.2–94.5%)	< 0.001
DP observed every 12 weeks	Rounds 1–3	49/3,486 (1.4%)	1.3%	91.9% (88.9–94.0%)	< 0.001
DP observed every 4 weeks		1/1,809 (0.06%)	0.1%	99.7% (97.7–100%)	< 0.001
None	Rounds 5–6	2/424 (0.5%)	0.4%	97.4% (89.7–99.4%)	< 0.001

DP = dihydroartemisinin–piperaquine.

* Adjusted prevalence for a child of average age (1.25 years).

† Including random effects to account for clustering within individuals and adjusted for study and age.

### Association between chemoprevention, IRS, and the prevalence of microscopic or submicroscopic parasitemia.

**Figure 4.**
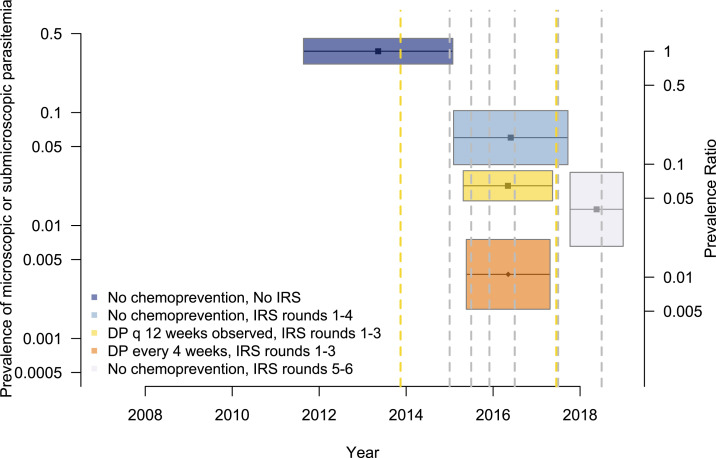
Adjusted prevalence of microscopic and submicroscopic parasitemia among participants from each intervention group. This analysis was limited to three studies that used sensitive methods to detect submicroscopic parasitemia (loop-mediated isothermal amplification/quantitative PCR). Each intervention is represented by a distinct colored box. The length of the boxes indicates the duration of the studies, and width indicates the 95% CI of the estimate. Prevalence ratios, relative to the reference group (no chemoprevention, no indoor residual spraying [IRS]), are shown as a secondary *y*-axis. Vertical dashed lines indicate the timing of population-level interventions: long-lasting insecticidal nets distribution (yellow) and rounds of IRS (gray). This figure appears in color at www.ajtmh.org.

This analysis was limited to data from 448 children from three studies. Among children who did not receive chemoprevention before the implementation of IRS (reference group), the crude and age-adjusted prevalence of microscopic or submicroscopic parasitemia at the time of routine visits were 47.3% and 34.9%, respectively (Table 4). In children who were not receiving chemoprevention, the first four rounds of IRS were associated with a relative reduction in the prevalence of 82.2% (95% CI: 69.5–90.3%, *P* < 0.001), and this increased to 96.0% (95% CI: 91.3–98.2%, *P* < 0.001) during the fifth and sixth rounds. In contrast to the results from the incidence and microscopic prevalence analyses, adding chemoprevention with DP given every 12 weeks was associated with additional reductions in the prevalence of microscopic and submicroscopic parasitemia as compared with IRS alone (relative reduction of 93.6%, 95% CI: 90.8–95.5%, *P* < 0.001). Addition of DP given every 4 weeks was associated with almost complete protection against microscopic or submicroscopic parasitemia (protective efficacy 98.9%, 95% CI: 97.8–99.5%, *P* < 0.001) (Table 4, Figure 4).

**Table 4:** Associations between intervention groups and the prevalence of microscopic or submicroscopic parasitemia

Intervention group		Crude prevalence	Adjusted prevalence*	Relative reduction† (95% CI)	*P*-value
Chemoprevention	Indoor residual spraying.				
None	None	178/376 (47.3%)	34.9%	reference group	
None	Rounds 1–4	20/233 (8.6%)	6.0%	82.2% (69.5–90.3%)	< 0.001
DP observed every 12 weeks	Rounds 1–3	117/3,462 (3.4%)	2.2%	93.6% (90.8–95.5%)	< 0.001
DP observed every 4 weeks		10/1,798 (0.6%)	0.4%	98.9% (97.8–99.5%)	< 0.001
None	Rounds 5–6	9/423 (2.1%)	1.4%	96.0% (91.3–98.2%)	< 0.001

DP = dihydroartemisinin–piperaquine.

* Adjusted prevalence for a child of average age (1.25 years).

† Including random effects to account for clustering within individuals and adjusted for study and age.

## DISCUSSION

Our results, from pooled analyses of cohort studies conducted over 11 years, showed that a combination of IRS and chemoprevention in the setting of high LLIN coverage and prompt and effective treatment of symptomatic malaria led to almost complete elimination of malaria in young children in the historically high-transmission area of Tororo district, Uganda. Children aged 0.5–2 years given only LLINs suffered almost five episodes of symptomatic malaria per year and during routine visits had almost 50% prevalence of malaria parasitemia based on sensitive molecular assays. Before the implementation of IRS, monthly DP, the most effective chemopreventive regimen studied, provided modest protection when administered unobserved at home. Remarkably, the implementation of IRS was associated with over 90% protection against symptomatic malaria and 80% protection against parasitemia during the first four rounds, with improving efficacy after two additional rounds. The addition of partially supervised chemoprevention with monthly DP to IRS was associated with almost complete protection against symptomatic malaria and parasitemia.

Long-lasting insecticidal nets and case management with artemisinin-based combination therapy (ACT) are key components of Uganda’s malaria control strategy and the only tools widely used throughout sub-Saharan Africa. In Tororo district, with high coverage of LLINs and prompt effective treatment with AL, the risk of severe malaria was extremely low, and there were no malaria-related deaths among young children over 1,200 person years of follow-up. However, even with these interventions, the burden of malaria was very high, implying that in this high-transmission setting, malaria elimination would not be feasible with LLINs and case management with artemisinin-based combination therapy (ACT) alone. We previously showed the proportion of houses in Tororo district with at least one LLIN was 78.5% in 2012, increasing to 95.5% in 2015 after the first LLIN campaign.^12^ In addition, all our cohort participants received a LLIN at enrolment. One factor that likely contributed to the persistently high burden of malaria following universal LLIN distribution was high-level pyrethroid resistance among mosquito vectors in Tororo district.^12^ By contrast, over a 22-year period at a historically high-transmission site in rural Senegal, changes in case management from chloroquine to an ACT followed by universal distribution of LLINs were temporally associated with dramatic declines in malaria burden, reaching pre-elimination levels.^9^

Historically, IRS has played a major role in the elimination of malaria in several countries outside Africa and in greatly reducing the burden of malaria in parts of Africa with low or seasonal transmission.^24,25^ Studies evaluating the benefit of adding IRS to LLIN distribution have yielded mixed results.^26–29^ Adding IRS to LLINs appears to have been most effective in areas where LLIN coverage was low and/or pyrethroid resistance high. In addition, in areas where LLIN coverage was high, adding IRS may have been most effective when using non-pyrethroid–based insecticides, likely explained by widespread resistance to pyrethroids, but not some other classes of insecticides available for IRS in Africa.^12^ In our setting, four years of sustained IRS first with carbamate and then organophosphate, in a context with universal LLIN coverage, was associated with a dramatic decrease in malaria burden to a threshold which would be considered pre-elimination.^3^ Our findings are similar to a report from the historically high-transmission island of Zanzibar, where a combination of interventions including ACTs for case management, universal LLIN distribution, and annual rounds of IRS was temporally associated with a marked reduction in malaria burden to pre-elimination levels between 2003 and 2015.^8^ Thus, IRS appears to have great promise for malaria control and potential elimination in high-transmission regions. However, although IRS has become a key component of Uganda’s malaria control strategy, resource constraints have limited its use to less than 10% of the country and necessitated its withdrawal in some areas.^30^ Currently, discussions on low-cost IRS delivery models that would allow scale up of this powerful intervention are ongoing in Uganda.^31^ Alarmingly, in areas of northern Uganda, the withdrawal of IRS in 2014 after five years of sustained use was followed by a rapid resurgence of malaria, despite universal LLIN distribution.^14^

Available results suggest that in high-transmission areas, limiting the use of antimalarial drugs to case management is unlikely to reduce the parasite reservoir sufficiently to achieve elimination in the absence of highly effective and sustained vector control.^10^ Chemoprevention, the regular use of antimalarial drugs to clear existing infections and prevent new infections, has been proposed to accelerate the path to elimination. Several countries in the sub-Sahel of West Africa have adopted a policy of seasonal malaria chemoprevention (SMC) with SP plus amodiaquine (AQ), which has been shown to be highly effective in reducing the burden of malaria in young children.^32^ However, SMC is not an attractive option for countries such as Uganda where transmission is perennial and resistance to SP is widespread. More recently, mass drug administration (MDA) has been investigated as a means of accelerating the path to elimination following the successful scale-up of traditional control interventions but with mixed results. Two rounds of MDA with DP were associated with a marked short-term reduction in the burden of malaria in southern Zambia,^33^ but two rounds of MDA with DP plus single low-dose primaquine were not associated with improvement in measures of malaria transmission in Zanzibar.^34^ Our findings show that highly effective chemoprevention with partially observed DP given monthly offered important benefits complementing those of IRS.

Our study had some limitations. First, our results were based on a pooled analysis across several studies and therefore subject to bias due to unmeasured confounders and/or secular trends unrelated to the interventions assessed. Indeed, malaria metrics have decreased across much of Africa in the last two decades, although not nearly to the extent observed in Tororo.^35^ Second, to maintain comparability across the various studies, we only included data from young children, and therefore cannot generalize our results to older age-groups. Indeed, a shift in the burden of malaria to older age-groups following declines in transmission intensity has been well described, and caution should be taken in extrapolating our findings to older children and adults.^8,9^ Third, submicroscopic parasitemia was assessed in only a subset of studies using different molecular methods, although the use of the more sensitive qPCR for the PRISM 2 study likely underestimated the impact of later rounds of IRS. Fourth, the independent impact of LLINs could not be estimated, as they were distributed among all study participants. Fifth, chemoprevention was only given to study participants, precluding our ability to evaluate the impact of this intervention on malaria transmission at the community level. Sixth, results obtained in the context of cohort studies may differ from, and in fact are likely superior to, those in a non-study situation, especially for chemoprevention, which requires full compliance with drug regimens for optimal efficacy. Seventh, in all studies, we used convenience sampling to enroll study participants, with the exception of the two PRISM studies, which enrolled children from households randomly selected from one subcounty in the district. The use of different sampling frames to enroll study participants could have been a source of bias. Finally, results obtained in eastern Uganda may not predict those observed in other high-transmission regions of Africa that may have different malaria transmission dynamics, drug sensitivity of malaria parasites, and insecticide sensitivity of mosquito vectors.

In summary, our findings suggest that malaria elimination in young children, the most vulnerable population, may be feasible in highly endemic African settings through the scale-up of available control interventions. However, in high-transmission settings that are characteristic of much of Uganda, marked reductions in the burden of malaria will likely require high coverage of multiple interventions for a sustained period. Prompt and effective treatment of symptomatic malaria and high LLIN coverage likely contributed to the low risk of severe malaria and absence of deaths due to malaria; however, marked reductions in malaria morbidity were only observed following the implementation of IRS. The addition of chemoprevention with monthly DP further accelerated declines in the burden of malaria, but even without chemoprevention, the burden of malaria was reduced to pre-elimination levels when the duration of IRS was extended beyond 3 years. These findings can serve as an important “proof of concept” and an encouragement that malaria elimination is a realistic goal for high-transmission African settings. Future research should include studies of how best to deliver interventions in real-world settings, considerations of cost-effectiveness, studies of when and how to scale back interventions without resulting in resurgence, and randomized trials to evaluate the impact of targeted or population-level established and new interventions.
